# Mate genetic similarity affects mating behaviour but not maternal investment in mice

**DOI:** 10.1038/s41598-023-37547-6

**Published:** 2023-06-29

**Authors:** Kerstin E. Auer, Jasmin Primus, Sonja Istel, Maik Dahlhoff, Thomas Rülicke

**Affiliations:** 1 Institute of in vivo and in vitro Models, University of Veterinary Medicine Vienna, Vienna, Austria; 2 Department of Biomedical Sciences, University of Veterinary Medicine Vienna, Vienna, Austria

**Keywords:** Sexual selection, Behavioural ecology

## Abstract

Maternal investment can affect the survival and development of offspring. Here we experimentally investigated in mice, whether females alter implantation rates and pup survival after embryo transfer depending on the genetic similarity with their vasectomised mating partner. We selected the MHC genotype and genetic background of males and paired females either with males that shared the same MHC haplotype and genetic background (CBA/J inbred males, isogenic group), that shared half of the MHC haplotype and genetic background (B6CBAF1 hybrid males, semi-isogenic group), or that had a different MHC haplotype and genetic background (C57BL/6N inbred males, allogenic group). We performed 304 pairings, resulting in 81 vaginal plugs, which confirmed mating. Plug rates were significantly higher in the semi-isogenic group (36.9%) compared to the isogenic group (19.5%), but not the allogenic group (26%). We found no difference in the number of implantation sites, the number of born or surviving pups until weaning, or litter weight or sex ratio between groups. Even though we found a mating bias, we found no difference in maternal investment under laboratory conditions. At least under pathogen-free conditions our study does not provide any evidence for differential maternal investment when females could increase offspring genetic diversity or heterozygosity.

## Introduction

Female mating preferences are well studied in various species, and there is ample evidence that female mate choice can influence female fitness^1–4^. Direct evidence that mate choice can increase female fitness comes from studies that compare the fitness of offspring from females that were experimentally mated with their preferred versus non-preferred males. For example, in house mice (*Mus musculus domesticus*), females paired with preferred males produced more litters and gave birth to offspring that were more viable^5^, and better able to survive an experimental infection^6^, compared to females that were mated with non-preferred males. These differences in offspring survival can be attributed to direct or indirect genetic benefits from mate choice. However, alternatively and not mutually exclusive, survival differences can also arise by differential maternal investment into offspring^7,8^.

Female reproductive investment should increase when females mate with males, which they perceive as more attractive or of high (genetic) quality^9^. This pattern is known as differential allocation^8–10^. In some cases, however, female reproductive investment has also been shown to increase when females mate with males that offer fewer benefits to offspring fitness. This phenomenon has been termed reproductive compensation^7,11,12^. Attributing differences in offspring viability to maternal effects is not simple, as effects arising from a male's heritable genetic quality, or other confounding paternal effects^13^ have to be discounted. Empirical evidence for differential maternal investment comes from studies in birds, fish, insects and mammals^5,9,14,15^ . However, most studies on differential allocation have considered mate quality in the context of good genes, i.e. assuming all females prefer the same males of high additive genetic quality^9,15^, and only a handful of studies have considered the influence of mate genetic diversity or genetic compatibility on maternal investment^16–20^.

The MHC (major histocompatibility complex) is a group of highly polymorphic genes that are crucial in host-parasite interactions^21^ and mate choice^22^. Increased MHC diversity or heterozygosity is expected to lead to increased disease resistance in offspring, and MHC-correlated mating preferences have been found in several mammalian species^22–32^. In these examples the MHC appears to bias mate choice through its effects on body odor and odor perception. In mice, females are known to discriminate between individuals with similar or dissimilar MHC genotypes, and female mating preferences are disassortative with regard to their MHC genotype^28,33^. Besides MHC mating preferences, female mice mate with multiple males and show a mating bias towards dominant, territorial males^34^. Under semi-natural conditions, when females are allowed to choose between two males, they actively engage in multiple mating^35,36^, thereby creating an arena for post-copulatory sexual selection and cryptic female choice.

MHC genes could play a role in cryptic female choice, either in leading to a selective fertilization^37^, and/or a selective abortion of offspring of certain males^38,39^. Studies on humans and rodents indicate that females are more likely to abort MHC-similar pregnancies^40,41^, and in vitro fertilizations are more likely to fail when couples share MHC alleles^42^. Moreover, couples that share MHC haplotypes have unusually long interbirth intervals, which could be explained by selective abortion^43^. Recently, a correlational study in horses found that females were more likely to become pregnant after instrumental insemination, when mares at the time of fertilization were exposed to stallions with more MHC dissimilar profiles^44^. Given that pregnancy rates were independent of the genotype of the actual sperm donor, this study suggests that females might selectively bias fertilization or implantation based on the genetic constitution of a perceived mating partner. However, we are not aware of any study in mammals that has experimentally tested whether male genetic similarity—genome-wide or at a specific locus—affects maternal investment in implantation rates and offspring development.

Differential maternal investment into offspring can be considered a form of post-copulatory mate choice. Surprisingly, the implication of differential maternal investment for sexual selection has received comparatively little attention, and its potential value for other research areas has not been considered. For example, studies on reproduction rarely consider evolutionary theory^45^. This lack of contact between the two fields is problematic, as sexual selection, and especially post-copulatory sexual selection can affect the evolution of male ejaculates, as well as female reproductive behavior and physiology^39^. Understanding female reproductive strategies and their investment into early pregnancies and gestation can improve reproductive outcomes, which can be beneficial for other research areas like reproductive medicine, the 3Rs in experimental use of laboratory animals, animal breeding, and species conservation^46^.

Here, we aimed to test whether females alter maternal investment depending on the genetic similarity of their mating partner. We paired females with males that either shared the same MHC haplotype and genetic background (isogenic), that shared half of the heterozygous MHC haplotype and genetic background (semi-isogenic), or that had a different MHC haplotype and genetic background (allogenic). We vasectomized males prior to pairing and performed embryo transfers in pseudo pregnant females to control for confounding effects arising though male or female genetic effects. We recorded mating and pregnancy rates, determined the number of implantation sites, and assessed pup survival until weaning. We expected that females were more attracted to males in the semi-isogenic or allogenic group compared to the isogenic group, and that embryo transfer rates and pup survival would be higher in these pairings, as females might invest more into offspring when they are paired with males that allow them to enhance offspring genetic diversity and heterozygosity, especially at the MHC^47^.

## Material and methods

### Experimental animals

All experimental mice were purchased from Janvier Labs, France. Animals were kept in same-sex groups in Typ IIL mouse IVC-cages (Tecniplast) equipped with wooden bedding (Lignocel 3–4 S, Rettenmaier and Söhne), nesting material (Pur-Zellin 4 × 5 cm; Paul Hartmann GmbH) and cardboard tubes (7.6 × 3.8 cm diameter, Special Diet Service) as enrichment. Commercial mouse diet (ssniff, V1534) and tab water were provided ad libitum and standard laboratory conditions (21 ± 1 °C temperature, 40–55% humidity,12:12 h light–dark cycle with lights on at 6:00 a.m.) were maintained before and during the experiment.

We used a pool of 120 CBA/J female mice as potential surrogate mothers and 11 CBA/J, 11 C57BL/6N and 11 B6CBAF1 hybrids (F1 from C57BL/6N females and CBA/J males) as vasectomized males for pairings. CBA/J females were purchased at the age of 6 weeks and kept in triplets until mating was performed at the age of 9 to 11 weeks. Males were purchased between 7 and 9 weeks and used at the age of 16 to 19 weeks for the experiment. In addition, we used 99 B6D2F1 hybrid females at the age of 8 to 11 weeks as oocyte donors and 11 B6D2F1 hybrid males at the age of 10 to 13 weeks as sperm donors for in vitro fertilization assays to generate embryos. We used F2 embryos as they show high genetic variability, thereby avoiding an implantation bias related to specific genetic interactions between surrogate mothers and embryos.

### Study design

We aimed to test whether females differentially invest into offspring survival and development depending on the genetic similarity of their potential sire. Therefore, we paired females with vasectomized males that either shared the same MHC haplotype and genetic background (isogenic), that shared half of the heterozygous MHC haplotype and genetic background (semi-isogenic), or that had a different MHC haplotype and genetic background (allogenic). To maximize potential effects of genetic similarity on maternal investment we simultaneously manipulated MHC and genome wide genetic similarity. In the isogenic group, CBA/J females were paired with CBA/J males. In the semi-isogenic group CBA/J females were paired with B6CBAF1 hybrid males and in the allogenic group the CBA/J females were paired with C57BL/6N males (see Table 1).

**Table 1:** Genetic background and MHC haplotype of recipient females and their vasectomized mating partners to induce pseudo pregnancy before embryo transfers.

Experimental group	♀ Background	♀ MHC haplotype	♂ Background	♂ MHC haplotype
isogenic	CBA/J	H2k	CBA/J	H2k
semi-isogenic	CBA/J	H2k	B6CBAF1	H2b/k
allogenic	CBA/J	H2k	C57BL/6N	H2b

We aimed to perform 25 embryo transfers in each experimental group. We performed odor stimulation in females to synchronize and time their estrus cycles by the Whitten effect^48^ before randomly assigning them to vasectomized males. After approximately 40 h, stimulated females were individually introduced to male home cages overnight and separated the following morning. We checked for the presence of a vaginal plug, which confirmed mating. Plug positive females were subsequently prepared for embryo transfers, unplugged females returned to the colony. Unplugged females that appeared to ovulate within 24 h (showing a swollen vulva, moist, and reddish vagina with large opening) were re-introduced to the same male’s cage in the evening. All other males were paired with a new female in the evening. Unplugged and unreceptive females were re-housed in same-sex groups for at least 5 days before being used for pairings again.

As we did not monitor mating behavior, we cannot exclude that mating occurred in the absence of a vaginal plug in females that appeared to ovulate soon. We re-introduced on average 26.5% of females (isogenic: 24.1%, semi-isogenic: 28.5% allogenic:26.9%). Approximately one third of the re-introduced females showed a mating plug and there was no apparent difference in the plug rate between groups (isogenic: 32.1%, semi-isogenic: 41.7% allogenic: 35.7%).

### Measurements of maternal investment

We recorded mating frequencies in the respective pairing groups and monitored females daily after embryo transfers to record pregnancies and births. We counted the number of offspring born and tracked pup development and pup loss on a daily basis. After 21 ± 1 days all remaining pups were weaned, and we determined individual pup body mass and sex. After weaning all female recipients (including those that did not give birth after embryo transfer) were sacrificed to determine the number of implantation sites by visual inspection of uteri after staining with 10% ammonium sulphide^49^.

### Embryo transfer

Embryo transfers were conducted over the course of three weeks. Every day, the order of embryo transfers was randomized. For transfers, females were anaesthetized by an intraperitoneal injection of ketamine/xylazine (ketamine: 100 mg/kg; xylazine: 4 mg/kg; Richter Pharma AG) and meloxicam (5 mg/kg Metacam; Böhringer Ingelheim-Vetmedica GmbH,) was administered subcutaneously for postoperative analgesia. Eyes were covered with eye ointment (Oleovit; Fresenius Kabi) and the skin around the surgery site was disinfected with 70% ethanol and rinsed with distilled water. Embryo transfers were conducted unilaterally into the right oviduct by a surgeon who was blinded for the pairing group. An incision of the unshaved skin and the peritoneum near the ovary was made to pull out the reproductive tract. The ovarian bursa was carefully ruptured, and sixteen 2-cell-stage embryos were transferred via the ovarian infundibulum into the ipsilateral ampulla of the uterine tube. Then, the reproductive tract was gently placed back into the abdominal cavity, the peritoneum sutured, and the skin closed with a Michel clamp. We performed surgery on a warmed table (37 °C) to prevent hypothermia. After waking up, animals returned to their home cage, and we checked animal wellbeing daily.

### In vitro fertilizations

We performed in vitro fertilization (IVF) assays to obtain 2-cell-stage embryos for embryo transfers. We performed IVF instead of flushing the oviducts of mated females, as this approach allowed us to control embryo environment and prevent potential effects from donor females. Donor females were super-ovulated by an intraperitoneal injection of 7.5 IU pregnant mare serum gonadotropin (PMSG, Folligon; Intervet), followed by 5 IU human chorionic gonadotropin (hCG, Chorulon; Intervet) 48 h later. Fifteen hours after the administration of hCG females were sacrificed by cervical dislocation to dissect the oviduct and to retrieve the cumulus oocyte complexes (COCs). COCs were transferred to a Petri dish containing a 200 µl drop of HTF medium covered with oil (NidOil™, Nidacon), and incubated at 37 °C and 5% CO_2_ until sperm was added.

Sperm donors were sacrificed before both *caudae epididymides* were extracted and transferred into a Petri dish containing a 90 μl drop of TYH medium covered with oil. We cut the epididymides open to enable sperm swim out before the tissue was removed and the sperm suspension was incubated for 60 min at 37 °C and 5% CO_2_ for capacitation. Afterwards 5 μl of the sperm suspension were added to the COCs and the IVF dish was incubated at 37 °C and 5% CO_2_ for fertilization.

After 4 h, we stopped the incubation of the IVF dish and washed all oocytes by transferring them through four 100 μl drops of HTF medium. We subsequently cultured vital cells overnight at 37 °C and 5% CO_2_ in a culture dish containing HTF. The next morning, we visually inspected cells and selected vital and evenly shaped 2-cell stages for embryo transfers.

### Vasectomy

Vasectomy was performed when males were 8 to 9 weeks old. Males were anaesthetized and treated for pain relief according to the same regime as used for embryo transfer. Once surgical anaesthesia was reached, the eyes were covered with eye ointment (Oleovit; Fresenius Kabi). Males were laid on their back and the abdomen was disinfected with 70% ethanol and rinsed with distilled water. Then, a longitudinal incision was made through the skin and the wall of the scrotum. The *vasa deferentia* were located and severed by cautery. The incision in the *tunica vaginalis* and the skin was then closed with sutures (Vicryl 5.0). Surgery was performed on a warmed table (37 °C) to prevent hypothermia. After surgery, animals returned to their home cage and daily health checks were performed.

After one week of recovery, we performed test matings to ensure that vasectomies were successful, and that the procedure did not affect male sexual behaviour. For test matings, each male was paired with a single, receptive female until a vaginal plug was detected. This treatment also ensured that all males were sexually experienced when used in the experiment.

### Data analysis

IBM SPSS Statistics 28 was used for statistical analysis. To test for differences in the probability of mating in females between the pairing groups we manually calculated Chi^2^ tests. To assess if pairing group affected the number of implantation sites, the number of pups born or weaned, litter weight at weaning, or sex ratio within litters we run linear mixed models (LMM) and included pairing group (isogenic, semi-isogenic, or allogenic) as fixed factor. Given that maternal body condition is expected to impact maternal investment into offspring^7^ we added female body mass at embryo transfer as covariate to the models. We further included male ID as random factor, as males were repeatedly used for mating trials, and data were non-independent. We confirmed that model assumptions were fulfilled in all models and transformed data if necessary.

### Ethical statement

Experimental procedures were discussed and approved by the Ethics and Welfare Committee of the University of Veterinary Medicine, Vienna and the national authority (Austrian Federal Ministry of Education, Science and Research) according to §§ 26ff. of the Animal Experiments Act, Tierversuchsgesetz 2012-TVG 2012 under license numbers BMBWF-68.205/0158-V/3b/2019 and GZ 2020-0.848.524 and performed in accordance with these regulations.

The reporting in this manuscript is in line with the recommendations in the ARRIVE guidelines^50^.

## Results

We performed 304 pairings between females and vasectomized males, resulting in 81 vaginal plugs with subsequent embryo transfers (Table 2). The likelihood of detecting a vaginal plug differed significantly between pairing groups (χ^2^ = 7.31, df = 2, p = 0.026), and plug rates were higher in the semi-isogenic than isogenic group (χ^2^ = 7.21, df = 1, p = 0.007). No difference in the number of vaginal plugs was found between the semi-isogenic and allogenic (χ^2^ = 2.61, df = 1, p = 0.106), or the allogenic and isogenic group (χ^2^ = 1.17, df = 1, p = 0.279).

**Table 2:** Number of pairings, resulting mating plugs and litters after embryo transfers in the different pairing groups.

Pairing group	Pairings (#)	Mating plugs (#)	Recipients /w implantations (#)	Litters (#)	Plug rate (%)	Pregnancy rate (%)	Birth rate (%)
Isogenic	116	23	21	21	19.8	91.3	91.3
Semi-isogenic	84	31	30	29	36.9	96.8	93.6
Allogenic	104	27	25	24	26.0	92.6	88.9

Pregnancy rate refers to the percentage of recipients that implanted at least one embryo and birth rate to the percentage of recipients that gave birth to at least one offspring after embryo transfer.

**Figure 1:**
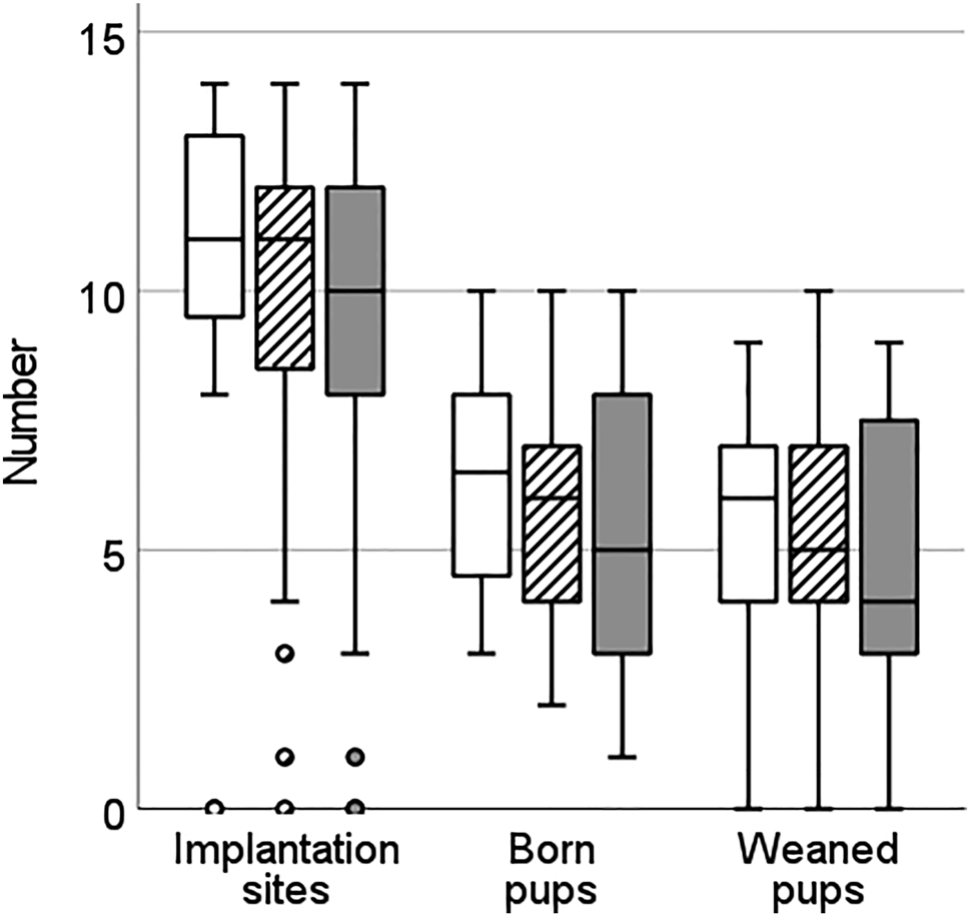
Boxplot of female reproductive investment in relation to male genetic similarity. Number of implantation sites, born and weaned pups after embryo transfer in females of the isogenic (white), semi-isogeneic (striped) or allogenic (grey) pairing group. Female recipients in all groups received 16 2-cell embryos each. Dot = mild outlier (Q1-1.5*IQ, or Q3 + 1.5*IQ).

We performed 81 embryo transfers (isogenic N = 23, semi-isogenic N = 31, allogenic N = 27) and the number of implantation sites after embryo transfers was on average 9.4, ranging from 0 to 14. However, the number of implanted embryos per female did not differ between pairing groups (LMM: F = 0.45, p = 0.645, Fig. 1). A total of 74 litters was born, and litter size at birth was on average 5.7, ranging from 1 to 10. The number of born pups per female after embryo transfer did not differ between pairing groups (LMM: F = 1.10, p = 0.338, Fig. 1), nor did the number of weaned pups (LMM: F = 0.53, p = 0.592, Fig. 1). Similarly, we found no effect of female body mass on the number of implantation sites (LMM: F = 1.54, p = 0.219), or the number of born (LMM: F = 0.38, p = 0.541) or weaned (LMM: F = 0.12, p = 0.732) pups.

**Figure 2:**
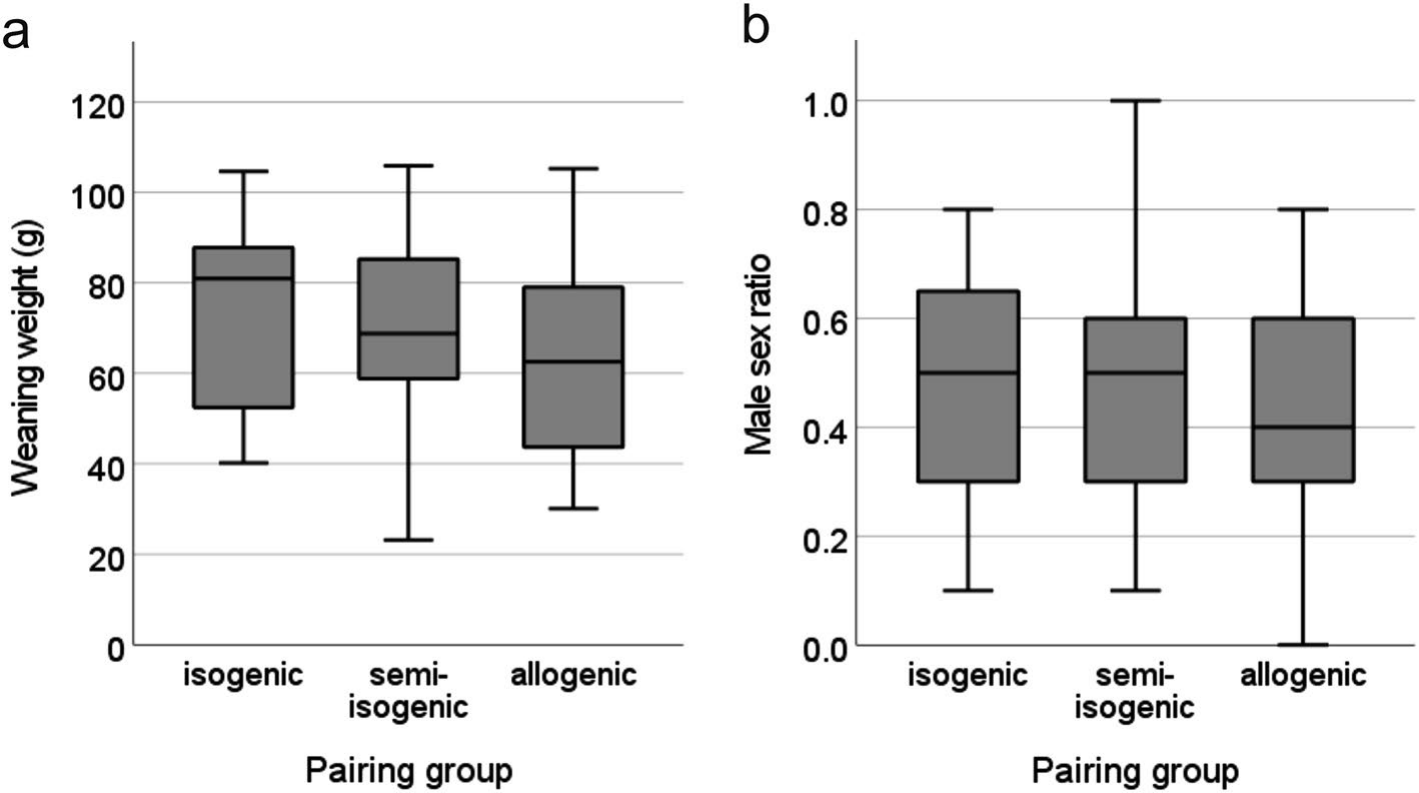
Boxplot of female reproductive investment in relation to male genetic similarity. (**a**) Litter weight at weaning and (**b**) male sex ratio in litters of females from the isogenic, semi-isogenic or allogenic pairing group.

Other parameters related to female investment like litter weight at weaning (LMM: F = 0.77, p = 0.476, Fig. 2a) and offspring sex ratio (LMM: F = 0.71, p = 0.503, Fig. 2b) did also not differ between experimental groups. However, we found that female body mass at embryo transfer was related to litter weight, and heavier females weaned significantly heavier litters on average (LMM: F = 8.57, p = 0.005). No relationship was found between female body mass and offspring sex ratio (LMM: F = 0.00, p = 0.986).

## Discussion and conclusion

We experimentally tested whether females adjust maternal investment into offspring depending on the genetic quality of their mating partner. Precisely, we manipulated the degree of genetic similarity between females and their mates, thereby altering the level of genetic benefits females could get for their offspring in terms of genetic diversity or heterozygosity.

We found highest mating rates in the semi-isogenic group, where females were paired with hybrid males, with which they shared half of their genome. Almost 37% of pairings in this group resulted in a mating plug, compared to 19.5% in the isogenic group, where both sexes originated from the same inbred strain, and 26% in the allogenic group, where females were paired with males from a different inbred strain. Hybrid mice are known for their hybrid vigor^51^, i.e. they show increased libido, improved disease resistance and higher fertility, and the significantly higher mating rate in the semi-isogenic than isogenic group could be explained by females being more attracted to such vital males. Hybrid males used in our study are produced by crossing C57BL/6N inbred with CBA/J inbred mice and are heterozygous at all loci for which their parents have different alleles. Given that males in the isogenic (CBA/J) and allogenic (C57BL/6N) group were both inbred and thus homozygous at almost all loci, our observed mating bias could also be explained by a female preference for heterozygous males, both at the MHC and genome wide. In wild mice, it has been shown that females are attracted to the scent of more heterozygous males and that this effect is stronger when females themselves are inbred^52^. Surprisingly though, only few studies have found support for this idea and most studies have failed to find statistically significant evidence that females prefer heterozygous males^53^.

Across various species, a positive relationship between individual heterozygosity at specific (e.g., MHC) or genome‐wide loci and various fitness traits has been found^53^. Thus, in theory, females can benefit from choosing a mate that will increase heterozygosity at some or many loci in their offspring^54^. However, as stated by Kempenaers, optimal mate choice will depend on whether the relationship between heterozygosity and fitness is linear (i.e., the more heterozygous, the better) or quadratic (i.e., individuals with intermediate levels of heterozygosity will have the highest fitness)^53^. We had no a priori prediction on whether females would be more attracted to males in the semi-isogenic or allogenic group, as females could gain genetic benefits for their offspring in both groups. Based on the observed mating plug rates in our study, females show a trend to preferentially mate with males that would allow them to optimize rather than maximize offspring heterozygosity. However, given that we did not investigate female mating preferences directly, the observed mating bias in this study could additionally, or alternatively be explained by males, if hybrid males are better in courting or coercing females into mating than inbred males. Whatever the underlying mechanisms, using hybrid males in assisted reproduction can elevate mating plug rates, thereby increasing the rate of pseudo pregnancy in females and reducing the number of animals required for the generation or rederivation of mouse lines when applying embryo transfer.

Even though we observed a mating bias, we did not find any evidence that females differentially invest into the survival or development of their offspring, as we found no difference in the number of implanted, born, or weaned pups, and no difference in the weight or sex ratio of weaned offspring depending on the genetic similarity between females and their mates. Given that we did not have a large enough sample size to exclude a type II error, we performed a power analysis to estimate the effect sizes, and the sample sizes that would be necessary to have an 80% chance to detect a potential effect of male genetic similarity on female implantation, birth or weaning rates. We found that effect sizes were small, ranging from f = 0.156 to f = 0.190, and would require sample sizes of 399 to 270 respectively to be able to detect a potential effect.

In mice, females could theoretically adjust maternal investment after copulation in physiologically inhibiting fertilization or implantation; and after pregnancy has occurred, females may abort fetuses or cannibalize newborn pups. In our study we performed embryo transfers to control for confounding effects arising from male or female genetic quality, and thus we could only test for differential maternal investment after the two-cell embryos had been transferred into the oviducts. It has previously been shown in mice that fertilization and the second meiotic division in the egg is not random with respect to the MHC haplotype of sperm^37^, and that this non-random fertilization and selective zygote development will lead to the production of more MHC heterozygous embryos when females were challenged with a viral infection^41^. These studies have two implications for the interpretation of our results:

First, in performing embryo transfers we might have missed any potential effects of differential maternal investment that occur prior to, or directly after fertilization. For example, females might adjust the number of ovulated oocysts in anticipation of the genetic similarity or compatibility of males. Second, females might only show differential maternal investment to improve offspring heterozygosity or genetic diversity under ecologically relevant conditions, i.e., when facing one or several pathogens in their environment. Mice in our experiment were kept under strict hygienic conditions and were free of all pathogens listed in the FELASA guidelines^55^. It has previously been shown that infected females adjust mate choice and can become more choosy when selecting a mate^56^.Thus, females might also selectively adjust maternal investment into offspring, if this increases the probability of producing disease‐resistant offspring. Future studies are required to assess how infection and pathogen-rich environments affect maternal investment when females can produce more disease-resistant offspring.

A recent study in horses showed that mares were more likely to become pregnant after instrumental insemination when they had previously been exposed to an MHC dissimilar than an MHC similar male^44^. Results from a follow up study, that investigated embryo survival in the oviduct, suggest that differences in pregnancy rates might rather be explained by selective embryo implantations than differential survival in the oviduct^57^. We found no evidence for differential implantations, even though mice have been shown to be capable of selectively blocking pregnancies in preventing implantations—at least in the context of infanticide avoidance^58^. Similar to our result, previous studies in mice also found no evidence that females would block pregnancies in within-strain matings, when genetically uniform offspring would be produced^59^, or alter mating behavior to increase offspring genetic diversity^36^.

Finally, we also found no evidence that females differentially invested into their offspring after giving birth. Infanticide was rare and weaning rates were above 90% in all three groups (see Table 3). We found a positive relationship between female body mass (which did not differ between pairing groups) and offspring weaning weights. This result is not surprising, as a strong link between a mother’s condition and her offspring can be expected given that gestation in mammals is one of the most energetically costly stages of reproduction for females^60^.

**Table 3:** Pup survival until weaning in the different pairing groups.

Pairing group	Litters (#)	Born pups (#)	Weaned pups (#)	Deceased pups (#)	Weaning rate (%)	Mortality rate (%)
Isogenic	21	126	116	10	92.1	7.9
Semi-isogenic	29	161	150	11	93.2	6.8
Allogenic	23	118	108	10	91.5	8.5

Weaning rate refers to the percentage of weaned pups and mortality rate to the percentage of deceased pups across all litters. In the allogenic group one litter with 8 pups had to be excluded from this analysis since the surrogate mother died after giving birth.

In conclusion, we experimentally tested whether females would increase maternal investment into the survival and development of offspring, depending on the genetic similarity of their mating partner at the MHC and genome wide. We vasectomized males prior to pairing and performed embryo transfers to control for confounding factors arising through male or female genetic effects. We found a mating bias in females for hybrid over same strain inbred males, indicating that females prefer to mate with more heterozygous males and avoid inbreeding. Alternatively, hybrid males could be better in courting or coercing females into mating. We found no evidence that females adjust maternal investment based on the genetic similarity of their mating partners. Our mice were kept under strict hygienic conditions and given that genetic benefits from mate choice are especially important under infection pressure, we cannot exclude that females might alter their investment when kept under more ecologically relevant conditions, or when challenged with a pathogen.

## Data Availability

The datasets supporting this article are available upon request from the corresponding author.
